# Comprehensive geriatric assessment in the emergency department: A protocol for a prospective cohort study

**DOI:** 10.12688/hrbopenres.13504.1

**Published:** 2022-04-01

**Authors:** Íde O'Shaughnessy, Katie Robinson, Margaret O'Connor, Mairéad Conneely, Fiona Steed, Damien Ryan, Leonora Carey, Denys Shchetkovsky, Elaine Shanahan, Aoife Leahy, Colin Quinn, Rose Galvin

**Affiliations:** 1 School of Allied Health, Faculty of Education and Health Sciences, Ageing Research Centre, Health Research Institute, University of Limerick, Limerick, Ireland; 2 Department of Ageing and Therapeutics, University Hospital Limerick, Limerick, Ireland; 3 School of Medicine, Faculty of Education and Health Sciences, University of Limerick, Limerick, Ireland; 4 Medicine Directorate, University Hospital Limerick, Limerick, Ireland; 5 Limerick EM Education Research Training (ALERT), Emergency Department,, University Hospital Limerick, Limerick, Ireland; 6 Department of Occupational Therapy, University Hospital Limerick, Limerick, Ireland

**Keywords:** Emergency Department, Older Adults, Frailty, Comprehensive Geriatric Assessment, Cohort Study

## Abstract

**Background: **Comprehensive geriatric assessment (CGA) has been shown to improve outcomes in hospitalised older adults; however, there is currently no compelling evidence to support CGA interventions within the Emergency Department (ED). The aim of this study is  to explore the clinical and process outcomes of older adults who receive ED-CGA over a period of six months after their initial ED attendance.

**Design: **Prospective cohort study.

**Methods: **The STrengthening the Reporting of the OBservational studies in Epidemiology (STROBE) standardised reporting guidelines will be adhered to. Older adults aged ≥65 years who score ≥2 on the Identification of Seniors at Risk (ISAR) tool and present to the ED with a medical complaint during the operational hours of the dedicated interdisciplinary team, will be considered eligible for recruitment. Demographic and health assessment information will be obtained at the ED index attendance followed by completion of an interdisciplinary CGA. A dedicated research nurse will complete follow-up telephone interviews with participants at 30 days and six months. The primary outcome will be incidence of hospital admission from the ED index attendance. Secondary outcomes will include functional decline, patient satisfaction with the ED index attendance, unscheduled ED reattendance(s), unscheduled hospital (re)admission(s), nursing home admission(s), healthcare utilisation, and death. Descriptive statistics will be used to profile the characteristics of the study participants and multivariate logistic and linear regression analysis will be used to analyse risk of adverse outcomes.

**Ethics and dissemination: **Ethical approval was granted by the University of Limerick Hospital Group Hospital Research Ethics Committee (107/2021). The authors will disseminate study findings through publication in a peer-reviewed journal and presentation at national and international conferences. Patient and public involvement will be sought from a panel of older adults at the Ageing Research Centre in the University of Limerick.

**Clinicaltrials.gov Identifier:** NCT05252182.

## Introduction

The significant growth in ED attendances is a growing public health issue ( Morley *et al.*, 2018), with attendances by older adults accelerating exponentially, beyond that due to population ageing alone ( Lowthian *et al.*, 2015). The most obvious causative factor for older adults increased aggregate demand for healthcare, specifically ED usage, is the clinical condition of frailty ( Clegg *et al.*, 2013). The presence of diminished homeostatic reserves leaves older adults more susceptible to acute exacerbations of comorbid and long-term conditions, which result in a concomitant increased demand for emergency and urgent care services ( Vermeiren *et al.*, 2016). There is unequivocal agreement within the literature that the ED is a challenging environment to deliver effective care to frail older adults ( Baum & Rubenstein, 1987). The complexity underlying the nature of their presenting complaint, which is often non-specific in nature may complicate ED care ( Salvi *et al.*, 2007) and investment in additional time and resources is frequently indicated to deliver a holistic assessment across multiple domains ( Limpawattana *et al.*, 2016). This can be challenging in the context of ED time-based targets, which have historically been designed for persons with single system illnesses and robust social networks ( Hommick *et al.*, 2016). Emergency medicine (EM) staff training has predominantly focused on clinically urgent and diagnostic specific complaints, thus creating a mismatch between the ED response and the changing demographic that they are increasingly facing ( Conroy & Turpin, 2016).

An ED attendance is often considered a sentinel event for an older adult ( Sanders *et al.*, 1996), with associated functional decline, increased risk of unplanned 30-day ED reattendance, and mortality well documented ( Lowthian *et al.*, 2016; Nagurney *et al.*, 2017; Shen *et al.*, 2018). Conversely, it affords clinicians an opportunity to stratify a high risk cohort followed by delivery of a holistic and bio-psychosocial intervention to mitigate against suboptimal clinical and process outcomes. These interventions include, but are not limited to, interface geriatrics and CGA, health and social care professionals (HSCP) and/or nursing interventions, and ED case management and post-discharge services referrals/liaison ( Cassarino *et al.*, 2019; Conroy *et al.*, 2011; Lowthian *et al.*, 2015). CGA is considered more effective than usual care in improving a range of outcomes for hospitalised frail older adults ( Ellis *et al.*, 2017). Evidence exists to support the feasibility of embedding CGA within the ED ( Conroy *et al.*, 2014) and an interdisciplinary model of care whereby clinicians integrate geriatric competencies into their practice has been recommended to meet the emergency needs of this patient population ( Conroy & Turpin, 2016).

Despite this recommendation, there is no robust high quality evidence that ED-CGAs are effective at improving clinical and process outcomes. Conroy *et al.* (2011) conducted a systematic review and meta-analysis of five randomised controlled trials (RCT), which evaluated the role of CGA in older adults being rapidly discharged from the ED or Acute Medical Unit. They found no compelling evidence to support implementation of CGA interventions in this patient cohort in terms of mortality [risk ratio (RR) 0.92, 95% confidence interval (CI) 0.55 to 1.52], readmissions [RR 0.95, 95% CI 0.83 to 1.08)], institutionalisation, functional status, quality-of-life or cognition. More recently, Harding (2020) provided an overview of five studies, which measured the impact of ED-CGAs on secondary healthcare utilisation, specifically 30-day ED reattendance and hospital admission. Two RCTs, one case matched cohort study, and two quasi-experimental pre and post intervention studies were reviewed. No definitive evidence was found to support the hypothesis that ED-CGAs can reduce ED reattendance or hospital admission 30 days post index attendance. We propose to explore the clinical and process outcomes of older adults who receive an interdisciplinary ED-CGA over a period of six months after their initial ED attendance through the conduct of a prospective cohort study.

## Methods

### Study design

The STROBE standardised reporting guidelines will be followed in the conduct and reporting of this prospective cohort study ( Von Elm *et al.*, 2007). Participant data collection and follow-up will take place between February 2022 to January 2023 (inclusive). 

### Ethics

Ethical approval for the study was granted by the Research Ethics Committee, Quality and Safety Department, University of Limerick Hospital Group Hospital (Ref: 107/2021). In accordance with the Data Protection Act 2018 (Section 36(2) Health Research Board, 2018), written informed consent will be obtained from eligible participants.

### Setting

The study setting will be the ED of University Hospital Limerick (UHL), which is a university teaching hospital with a large catchment area in the Mid-West of the Republic of Ireland. UHL is the central hub for a larger hospital group with six hospital sites, all functioning as a single hospital system caring for a substantially rural population of approximately 400,000. The university teaching hospital is the only hospital in the group that has a 24/7/365 emergency care and critical care service and has 455 inpatient beds. 76,667 ED attendances were recorded in 2021 of which 19,901 were older adults aged ≥ 65 years.

### Population of interest

Older adults aged ≥ 65 years who present to the ED of UHL (Monday-Friday, 08:00-16:00) between February 2022 and July 2022 will be deemed eligible for recruitment provided they meet the following criteria:

***Inclusion criteria***. We will include older adults aged ≥ 65 years who have a Manchester Triage System (MTS) category of 2 to 5 ( Mackway-Jones, 1997) and are presenting with a medical complaint. Eligible participants will be identified by a member of the interdisciplinary ED-CGA team and must screen positive for risk of adverse outcomes on the ISAR tool. The ISAR is composed of six simple yes/no items; a score of ≥ 2 indicates that the older adult is at increased risk of adverse outcome following ED index presentation ( McCusker *et al.,* 1999).

***Exclusion criteria***. Exclusions to recruitement will apply where older adults are deemed not to have decision-making capacity to provide informed consent or if attendance to the ED is outside of the operational working hours of the interdisciplinary ED-CGA team. Those presenting with acute cardiac and/or neurological pathology; injuries requiring surgical intervention; or high illness acuity, which necessitates treatment in the resuscitation room throughout their ED attendance, will be excluded.

### Recruitement and data collection

***ED index attendance***. A dedicated research nurse or clinical member of the interdisciplinary ED-CGA team will provide prospective participants with an information leaflet and explain the objective of the study. If the participant agrees to take part, he/she will be asked to read and sign a consent form. After consenting to recuitement, each participant will undergo a baseline assessment inclusive of a demographic questionnaire and a health assessment. Demographic information will include participant’s gender, age, marital status, residential status (living alone, living with others, nursing home resident, other), ethnicity, socioeconomic status (level of eduction, past/present occupation), mode of arrival to the ED (private transport, ambulance, public transport, other), source of referral (self-referral, general practitioner, out of hours general practitioner, injury unit, nursing home, etc), index complaint and triage category as per the MTS ( Mackway-Jones, 1997). The health assessment will comprise of the following measurements: the 21-item Charlson Comorbidity Index score ( Charlson *et al.*, 1994) to profile baseline comorbidities; a list of medications; a global measure of functional ability through the self-rated Barthel Index (BI) for activities of daily living ( Mahoney & Barthel, 1965); delirium and cognitive screening through the 4AT ( Bellelli *et al.*, 2014); health-related quality of life through the EuroQoL survey 5-dimension and 5-levels form (EQ-5D-5L) ( Rabin & de Charro, 2001); frailty status through Rockwood’s Clinical Frailty Scale ( Rockwood *et al.*, 2005); and nutritional status through the Malnutrition University Screening Tool (MUST) ( Elia & Russell, 2009).

Following baseline assessment, participants will undergo an interdisciplinary CGA across multiple domains including, but not limited to, medical, functional, cognitive, and psychosocial abilities ( Rubenstein *et al.*, 1991). The interdisciplinary team will compromise a registrar in geriatric medicine, specialist geriatric nurse, senior occupational therapist, senior physiotherapist, and senior medical social worker with dual governance from consultants in EM and Geriatric Medicine. Members of the team will be guided by their clinical expertise and competencies, scope, and codes of professional practice. Individualised care planning and referrals to out-of-hospital pathways, as appropriate will be the forefront of the teams’ practice. Where older adults are referred to specialist geriatric ambulatory care hubs, the focus will be on completing the priortity domians and components of CGA in the ED with timely completion of the assessment in its entirety in the specialist hubs, within seven days of referral.

***Follow-up assessment***. The research nurse will complete follow-up telephone interviews with participants at 30 days and six months. He/she will complete the 18-item Patient Satisfaction Questionnaire ( Marshall & Hays, 1994) at 30-day follow-up to explore participants’ satisfaction with their ED attendance. The BI ( Mahoney & Barthel, 1965) and EQ-5D-5L ( Rabin & de Charro, 2001) will be completed with participants in addition to a questionnaire, which will look specifically at capturing healthcare utilisation e.g. geriatric ambulatory care services, GP visits, public health nurse visits, home care support, outpatient clinic attendance, HSCP input etc. The questionnaire will follow the same structure and outline as per the prospective cohort study by Leahy *et al.* (2020). Data on objective measures such as unscheduled ED reattendance, hospital (re)admission(s), nursing home admission, and death will be ascertained from routine hospital data. Withdrawals and participants lost to follow-up will be recorded.

### Outcome variables

The primary outcome measure will be incidence of hospital admission from the ED index attendance. Secondary outcomes include functional decline (including functional decline at discharge among the admitted cohort), patient satisfaction with the ED index presentation, incidence of unscheduled ED reattendance(s), unscheduled hospital (re)admission(s), nursing home admission(s), healthcare utilisation, and death within 30 days and six months of the ED index attendance.

### Sample size

Our study is not hypothesis driven; therefore, formal power calculations are not applicable. All prospective participants that meet inclusion criteria will be invited to participate.

### Statistical analyses

Pseudonymised data will be stored on an encrypted and password protected electronic data capture system (CASTOR). Members of the research team who have responsibility for entering pseudonymised participant data, will have their own unique log in and password for the system. Hard copies of baseline and follow-up quesionnaires and consent forms will be stored in a locked cabinet in an office with restricted access.

Descriptive statistics will be used to profile the baseline characteristics of the cohort. Categorical measures (e.g., rates of 30-day ED reattendance) will be analysed in terms of frequencies and percentages; continuous measures (e.g., Barthel Index) will be analysed in terms of mean and standard deviation for normally distributed data and as median and interquartile ranges for non-parametric data. Multivariate logistic and linear regression analysis will be used to analyse risk of adverse outcomes and we will report adjusted RR or beta coefficients with 95% CI. Anonymised data generated will be published in an open access repository with an associated data dictionary

### Dissemination

The authors will disseminate study findings through publication in a peer-reviewed journal and presentation at national and international conferences. A lay summary of findings will be presented to the Patient and Public Involvement (PPI) panel of older adults that has been established at the Ageing Research Centre in the University of Limerick ( Conneely *et al.*, 2020). The focus of this session will be to discuss the findings with this group so that the discussion section of the paper can integrate their views and opinions.

### Study status

Data collection and follow-up is ongoing. Study completion is expected in January 2023.

## Discussion

EDs worldwide are challenged by an exponential growth in attendances, many of whom are frail older adults who have higher burdens of chronic diseases and multiple comorbidities. It is clear that older adults’ distinctive and multifactorial care needs are not suited to an episodic healthcare system designed around a single system illness. Health services and models of care must be adapted to meet the needs of an ageing international demographic. Further high quality evidence is required to support the implementation of cost-effective and holistic models of care that are responsive to the needs of older adults within the ED.

This study will adopt a longitudinal approach to exploring older adults’ outcomes following ED attendance, which will have relevance for clinicians, policymakers, and funders. Consideration of patient care as a continuum, rather than discrete encounters, has the potential to lead to identification of effective collaborative strategies and thus an integrated approach to care. The outcome variables included in the study will include a range of clinical, patient-reported and process outcomes. Inclusion of patient reported outcomes measures will assist clinicians in re-focusing care around older adults’ priorities and preferences.

## Data availability

### Underlying data

No data are associated with this article.
