# Supplementary material for: Comprehensive geriatric assessment in the emergency department: A protocol for a prospective cohort study
Source: HRB Open Res. 2022 Sep 14;5:26. Originally published 2022 Apr 1. [Version 2] doi: 10.12688/hrbopenres.13504.2 (PMC9582574; doi:10.12688/hrbopenres.13504.2)
Supplement: Peer review history

# Peer review: Reviewer response for version 2

**DOI:** 10.21956/hrbopenres.14888.r32828

**Published:** 2022-10-19

**Authors:** Kirstyn James

**Affiliations:** 1 Cork University Hospital, Cork, Ireland

Thank you to the authors for addressing all comments. The revised manuscript is well written and I recommend approving this manuscript.

Is the study design appropriate for the research question?

Yes

Is the rationale for, and objectives of, the study clearly described?

Yes

Are sufficient details of the methods provided to allow replication by others?

No

Are the datasets clearly presented in a useable and accessible format?

Not applicable

Reviewer Expertise:

Geriatric Medicine, frailty

I confirm that I have read this submission and believe that I have an appropriate level of expertise to confirm that it is of an acceptable scientific standard.

# Peer review: Reviewer response for version 1

**DOI:** 10.21956/hrbopenres.14731.r32665

**Published:** 2022-08-24

**Authors:** James van Oppen

**Affiliations:** 1 Department of Health Sciences, University of Leicester, Leicester, UK

Thank you for sharing this protocol for your study. I understand from the methods that initial recruitment will be complete by now.

 The protocol serves to answer an important knowledge deficit. In particular the study is strengthened by the use of PROMs to measure a person-centred perspective. To my knowledge, this will be the first large scale evaluation of ED-CGA to represent the patient voice in this way, rather than focussing only on service metrics.

 Good luck with the analyses and I will look forward to reading your findings.

Is the study design appropriate for the research question?

Yes

Is the rationale for, and objectives of, the study clearly described?

Yes

Are sufficient details of the methods provided to allow replication by others?

Yes

Are the datasets clearly presented in a useable and accessible format?

Not applicable

Reviewer Expertise:

Healthcare outcome measures in Geriatric Emergency Medicine

I confirm that I have read this submission and believe that I have an appropriate level of expertise to confirm that it is of an acceptable scientific standard.

# Peer review: Reviewer response for version 1

**DOI:** 10.21956/hrbopenres.14731.r32063

**Published:** 2022-05-31

**Authors:** Kirstyn James

**Affiliations:** 1 Cork University Hospital, Cork, Ireland

O’Shaughnessy *et al.* present the protocol for a cohort study investigating the association between comprehensive geriatric assessment (CGA) interventions in older adults and hospital admissions from the Emergency Department (ED).

 The primary outcome variable in this study is hospital admissions at the index ED attendance. The explanatory variable in this study is comprehensive geriatric assessment. The study will also explore the relationship between CGA interventions and functional decline, patient satisfaction and death.

 This study is well designed and has potential to contribute to the knowledge base in the field of frailty and geriatric emergency medicine. I have a few points for the authors to consider.

**Major points:**

*Material & Methods*

1. Sample size: The authors state that this study is not hypothesis driven. However, there is a clearly defined outcome and explanatory variable. The authors also plan to draw inferences on their findings using multivariate linear and logistic regression analyses. Can the authors please elaborate further on why a power calculation is not possible in this study?
2. The intervention in this study is inter-disciplinary CGA. Can the authors provide any additional information as to what types of interventions will be used e.g. deprescribing, strength training, referral for corrective glasses? This will be important information to allow replication of this study design.

Is the study design appropriate for the research question?

Yes

Is the rationale for, and objectives of, the study clearly described?

Yes

Are sufficient details of the methods provided to allow replication by others?

No

Are the datasets clearly presented in a useable and accessible format?

Not applicable

Reviewer Expertise:

Geriatric Medicine, frailty

I confirm that I have read this submission and believe that I have an appropriate level of expertise to confirm that it is of an acceptable scientific standard, however I have significant reservations, as outlined above.

# Response

**Published:** 2022-09-06

**Authors:** Íde O'Shaughnessy

**Affiliations:** University of Limerick, Ireland

Thank you for your comments. Based on your feedback, we have completed a power calculation, which is outlined in the 'Sample size' subsection of the manuscript.

 With respect to the interdisciplinary CGA intervention(s), we have inserted a description of typical interventions delivered by the team.
